# Computed Tomography Angiography in Microsurgery: Indications, Clinical Utility, and Pitfalls

**Published:** 2013-08-07

**Authors:** Gordon K. Lee, Paige M. Fox, Jonathan Riboh, Charles Hsu, Sepideh Saber, Geoffrey D. Rubin, James Chang

**Affiliations:** Division of Plastic and Reconstructive Surgery, Stanford University Medical Center, Stanford, Calif

## Abstract

**Objective:** Computed tomographic angiography (CTA) can be used to obtain 3-dimensional vascular images and soft-tissue definition. The goal of this study was to evaluate the reliability, usefulness, and pitfalls of CTA in preoperative planning of microvascular reconstructive surgery. **Methods:** A retrospective review of patients who obtained preoperative CTA in preparation for planned microvascular reconstruction was performed over a 5-year period (2001–2005). The influence of CTA on the original operative plan was assessed for each patient, and CTA results were correlated to the operative findings. **Results:** Computed tomographic angiography was performed on 94 patients in preparation for microvascular reconstruction. In 48 patients (51%), vascular abnormalities were noted on CTA. Intraoperative findings correlated with CTA results in 97% of cases. In 42 patients (45%), abnormal CTA findings influenced the original operative plan, such as the choice of vessels, side of harvest, or nature of the reconstruction (local flap instead of free tissue transfer). Technical difficulties in performing CTA were encountered in 5 patients (5%) in whom interference from external fixation devices was the main cause. **Conclusions:** This large study of CTA obtained for preoperative planning of reconstructive microsurgery at both donor and recipient sites study demonstrates that CTA is safe and highly accurate. Computed tomographic angiography can alter the surgeon's reconstructive plan when abnormalities are noted preoperatively and consequently improve results by decreasing vascular complication rates. The use of CTA should be considered for cases of microsurgical reconstruction where the vascular anatomy may be questionable.

Choosing appropriate vessels for microvascular anastomosis can be challenging in many cases. The ideal vessels should be widely patent, should be of sufficient size and caliber, should possess adequate blood flow, and should not be traumatized. Evaluation of the vasculature at both donor and recipient sites may be performed through clinical examination, radiological studies, or direct surgical exploration. Ideally the surgeon would want to determine the feasibility of the planned microvascular anastomosis, anticipate the likelihood of flap success, and evaluate any vascular abnormalities that might require modification of the operative plan. Currently, there is no consensus as to the optimal method to reliably evaluate the vascular anatomy prior to microsurgical reconstruction.

Digital subtraction angiography (DSA) is considered by some to be the criterion standard for answering these questions.^1^ However, DSA involves risks of arterial puncture, pseudoaneurysm, bleeding, and renal injury from intravenous contrast.^2^ Therefore, alternative and considerably less invasive techniques, such as magnetic resonance angiography (MRA),^3^ duplex ultrasonography,^4^ and computed tomography angiography (CTA),^2^ are often used. Regardless of the method, preoperative vascular imaging has proven useful across the spectrum of microsurgical reconstruction—from the extremities to the head and neck region.^5^^-^7 We define clinical utility in this context as the ability of an imaging modality to reliably detect vascular anomalies, directly influence surgical planning, and ultimately improve patient outcome.

Among the various imaging modalities, CTA has emerged as a potentially useful tool that exhibits several important advantages. It provides high-resolution images of both arterial and venous systems and allows for simultaneous 3-dimensional reconstructions of adjacent bones and soft tissue.^8^ Furthermore, there is preliminary evidence that CTA may safely and reliably replace DSA as a primary method of vascular investigation.^9^^,^^10^ Computed tomographic angiography has been previously used to evaluate blood vessels in cases of extremity trauma and flap preparation.^2^^,^^11^^-^15

We hypothesized that obtaining reliable delineation of vascular anatomy at the donor and/or recipient site through preoperative CTA could significantly influence the original microsurgical plan by assisting the surgeon in the selection of the best recipient vessels or the best flap to use. Therefore, the purpose of our study was to determine the safety, accuracy, and clinical utility of CTA in the preoperative planning of reconstructive microsurgery.

## METHODS

We obtained institutional review board approval and performed a retrospective analysis on all patients who underwent a preoperative CTA in preparation for microsurgical reconstruction after at a single tertiary care academic institution over a 5-year period (2001–2005). Patients who underwent CTA for any indication other than microsurgical planning were not included in the study. Patients obtained CTA routinely for cases of free fibula flap donor site evaluation; however, for other scenarios CTA was obtained on the basis of clinical judgment by the surgeon in which there was a concern for abnormal vascular anatomy. Computed tomographic angiography was obtained using a 16-row detector scanner (Lightspeed; GE Medical Systems, Waukesha, Wis). Our institution's previously published CTA protocol was followed in this study.^2^^,^^16^

We determined the original plan for microsurgical reconstruction based on review of physicians’ notes that also indicated when an alternative therapy was chosen subsequent to CTA findings. Abnormalities were identified upon review by both the radiologist and the surgeon. Abnormalities included vascular occlusion, stenosis, and atherosclerotic lesions but did not include normal anatomic variants. We evaluated defect type, choice of flap, vessel image quality, influence of CTA on operative planning, concordance with intraoperative findings, CTA-related complications, and flap-related complications. Intraoperative evaluation of blood vessels was considered the criterion standard in our analysis.

Standard statistical analysis was performed to calculate the following: sensitivity (number of abnormal CTA correlating with actual intraoperative abnormality divided by number of actual intraoperative abnormality); specificity (number of normal CTA correlating with normal intraoperative anatomy divided number of normal intraoperative anatomy); positive predictive value (number of abnormal CTA correlating with actual intraoperative abnormality divided by number of abnormal CTA); and negative predictive value (number of normal CTA correlating with normal intraoperative anatomy divided by number of normal CTA).

## RESULTS

### Patient demographics

A total of 94 patients underwent CTA as part of the preoperative workup for microvascular reconstruction during the study period. There were 55 men (59%) and 39 women (41%), with an average age of 39 years (range: 3–78 years) (Table 1). Computed tomographic angiography was performed on the upper extremity (n = 21, 22%), lower extremity (n = 34, 36%), free fibula donor site (n = 17, 18%), head and neck (n = 8, 9%), breast and chest wall (n = 13, 14%), and abdomen (n = 1, 1%) (Table 2). Computed tomographic angiography imaging of the upper and lower extremities (with the exception of the free fibula donor site) typically followed traumatic injury, and CTA of the head and neck typically preceded oncologic reconstruction. Recipient vessels were imaged before autologous breast or chest wall reconstruction. The abdomen was imaged in anticipation of abdominal reconstruction in one case. Of the 94 patients, 73 (78%) patients underwent intraoperative exploration of the vascular site in question, whereas in 20 patients (21%) the vessels were not explored as this was not required for the operation.

### Image quality

Computed tomographic angiography produced digital reconstruction of complex 2-dimensional and 3-dimensional images of vasculature and surrounding soft tissue. In 89 patients (95%), the images obtained were of excellent quality (as deemed by the surgeon and radiologist) and allowed for the detection of abnormalities ranging from mild vessel stenosis to complete obstruction. In 5 patients (5%), the vascular anatomy could not be fully delineated by CTA: in 3 cases (3%), this was due to artifact from external metal fixators, in 1 case (1%) due to excessive venous flow obscuring the arterial phase, and in 1 case (1%) due to extensive soft tissue distortion (Table 3).

### Clinical utility of CTA

Clinical utility was gauged by assessing the proportion of operative plans changed by CTA results across the breadth of microsurgical procedures. In our series of 94 patients, 48 scans (51%) revealed a vascular abnormality such as perivascular fibrosis, vessel narrowing, or total vessel occlusion. In 42 cases (45%), the original operative plan was changed because of abnormal CTA findings. In other words, out of 48 patients with abnormal CTA findings, 42 cases (88%) resulted in a change of operative plan (Table 2). In the remaining 6 cases (13%), the CTA abnormalities were rated as minor, age-related perivascular disease and did therefore not affect the operative plan.

Preoperative CTA led to a change in operative planning after evaluation of recipient vessels in the upper extremity (86%) and lower extremity (29%), assessment of the fibula as a free flap (41%), and evaluation of recipient vessels in the head and neck (38%), breast and chest wall (23%), and abdomen in one case (100%) (Table 2). Overall, operative plans were changed in 41% of donor site evaluations and in 45% of recipient site evaluations (Table 2). Abnormalities noted at the donor site resulted in a change of operative plan 100% of the time, while abnormalities noted at the recipient site led to a change of operative plan 85% of the time (Table 2). Of note, traumatic injuries correlated with a higher incidence of radiologic abnormalities (55%), followed by infection (45%) and cancer (43%) (Table 2).

### Concordance with intraoperative findings

Intraoperative visualization of vascular anatomy was considered the criterion standard against which to validate CTA findings. In 73 patients (78%), paired descriptions of vascular anatomy from both CTA and intraoperative notations were available for review, and our analysis of these cases showed that CTA was highly reliable. Indeed, preoperative CTA was found to have a sensitivity of 94%, specificity of 97%, positive predictive value of 97%, and negative predictive value or 95% (Table 3).

### Complications and limitations

All 94 patients were followed for a mean of 12 months (range: 1–58 months). There were no cases of CTA-related or intravenous contrast-related complications in this study.

There were 7 total patients with external metal fixators in our study, and in 5 of these cases (71%), it was difficult to interpret the vascular anatomy from CTA. In 3 of these cases (43%), this was due to interference from external fixation, while in the other 2 cases, this was due to excessive venous flow obscuring the arterial phase or due to extensive soft tissue distortion (Table 3).

## CASE REPORTS

### Case 1: CTA in breast reconstruction

**Figure 1:**
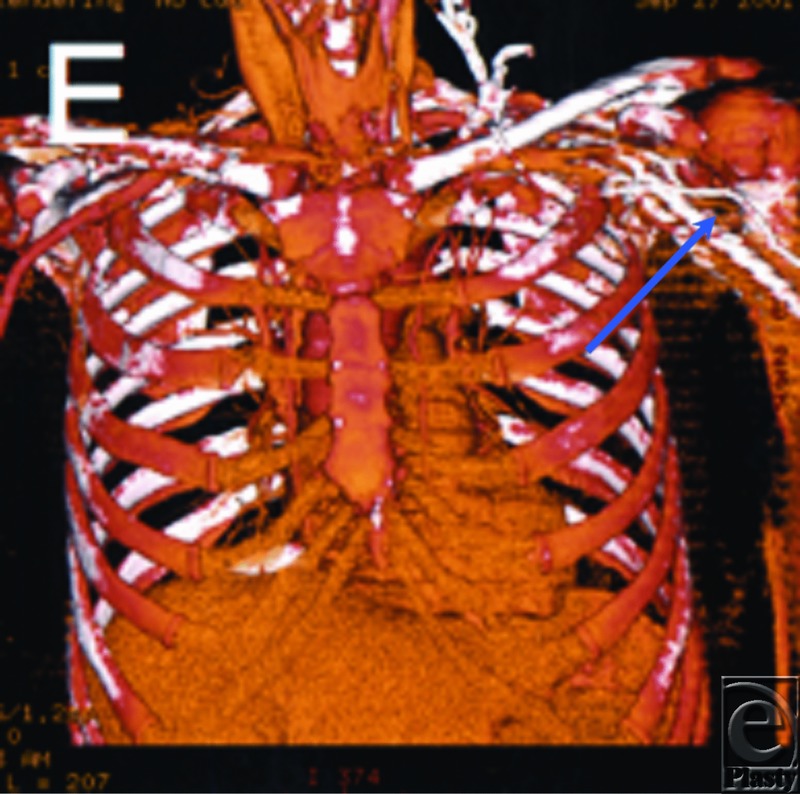
CTA in reconstructive breast surgery. CTA imaging of thoracodorsal vessels to assess suitability of the vessels as recipients for a DIEP flap. The vessels were patent, but embedded in large amounts of surgical scar, with marked perivascular fibrosis. DIEP indicates deep inferior epigastric perforators.

A 58-year-old woman presented for delayed autologous left breast reconstruction after mastectomy. A CTA was obtained to confirm the suitability of the thoracodorsal vessels as recipients for free tissue transfer. The vessels were patent but embedded in large amounts of surgical scar, with marked perivascular fibrosis (Fig 1). As a result, the operative plan was changed and a pedicled TRAM (transverse rectus abdominus myocutaneous) flap was performed successfully (as the patient did not desire rib resection for internal mammary vessel exposure).

### Case 2: CTA in upper limb reconstruction

**Figure 2:**
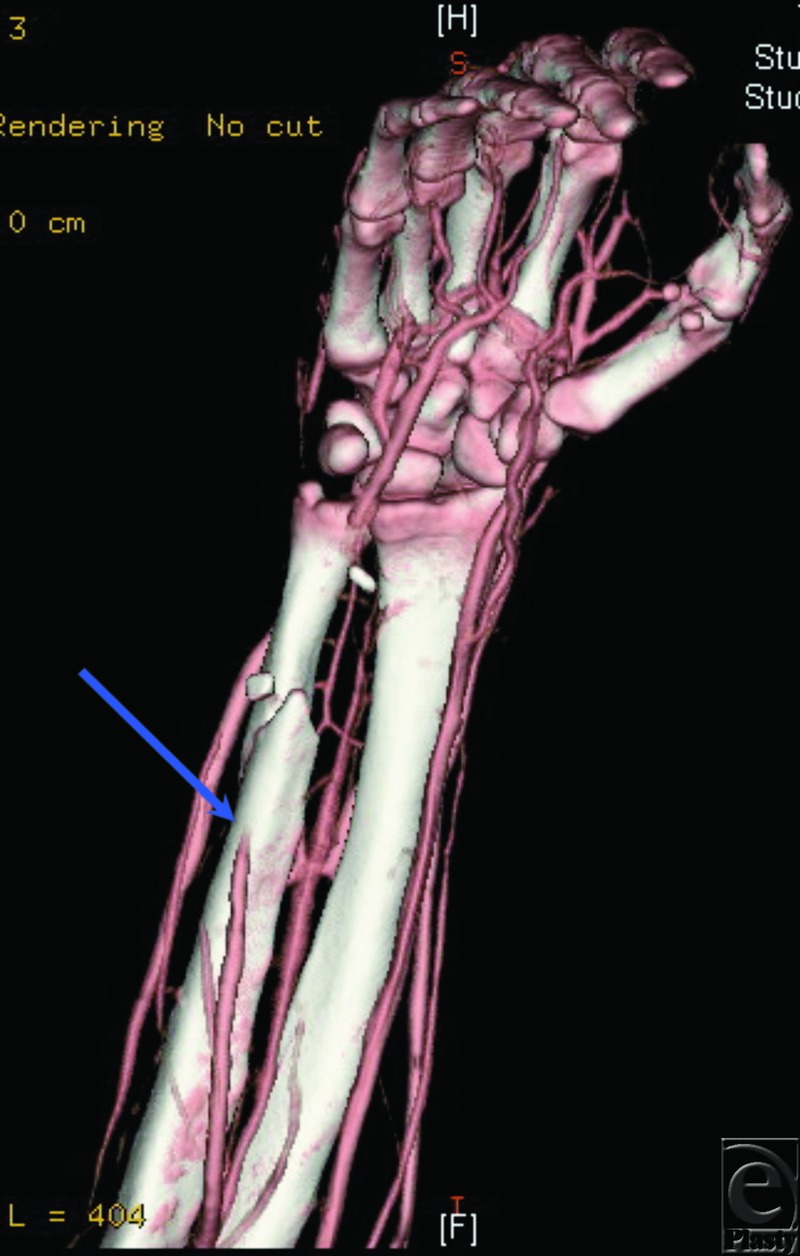
CTA in reconstruction of the upper limb. CTA of the right upper extremity demonstrating occlusion of the ulnar artery in the distal forearm at the level of the fracture site. The hand is supplied through the radial artery and a complete palmar arch.

A 19-year-old man suffered a gunshot wound to the right hand and wrist, requiring a free flap to provide soft tissue coverage. Examination of the patient's distal pulses was limited by significant swelling and pain. A preoperative CTA demonstrated occlusion of the ulnar artery in the distal forearm (Fig 2). The hand was supplied through the radial artery and a complete palmar arch. The patient thus successfully underwent free rectus flap reconstruction with microvascular anastomosis to the ulnar artery proximal to the site of occlusion.

### Case 3: CTA in lower limb reconstruction

**Figure 3:**
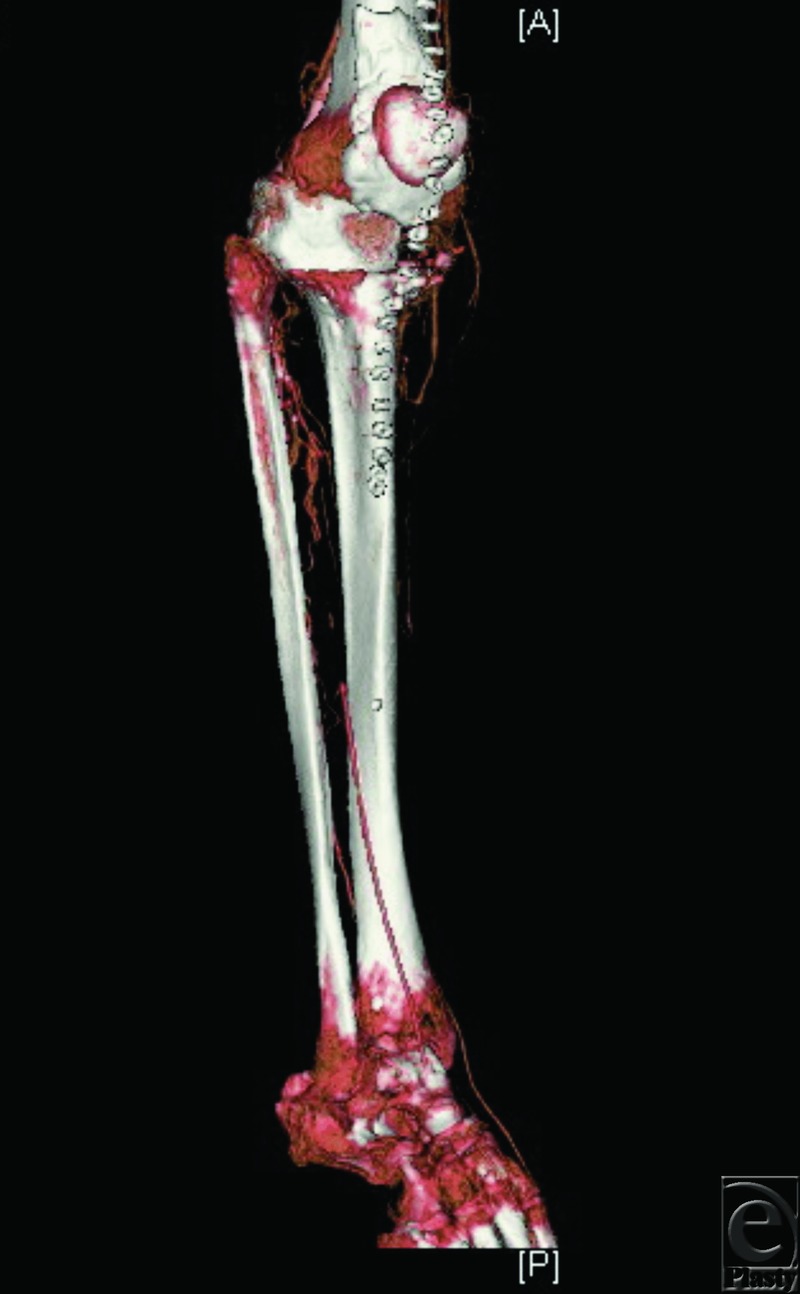
CTA in reconstruction of the lower limb. CTA of a traumatized right lower extremity demonstrating severe atherosclerotic disease as well as numerous focal occlusions of the anterior tibial and posterior tibial arteries distal to the trifurcation of the popliteal artery.

An 82-year-old woman was considered for free flap coverage of a chronic and large open wound over her right knee after removal of an infected knee prosthesis. A CTA of the right anterior and posterior tibial arteries showed numerous focal occlusions distal to the trifurcation of the popliteal artery. Thus, the dominant blood supply to the foot was from the peroneal artery (Fig 3). Free tissue transfer was felt to be risky due to the lack of suitable recipient vessels. Instead, medial and lateral gastrocnemius flaps and a split-thickness skin graft were used to successfully cover the defect.

### Case 4: CTA of recipient site in head and neck reconstruction

**Figure 4:**
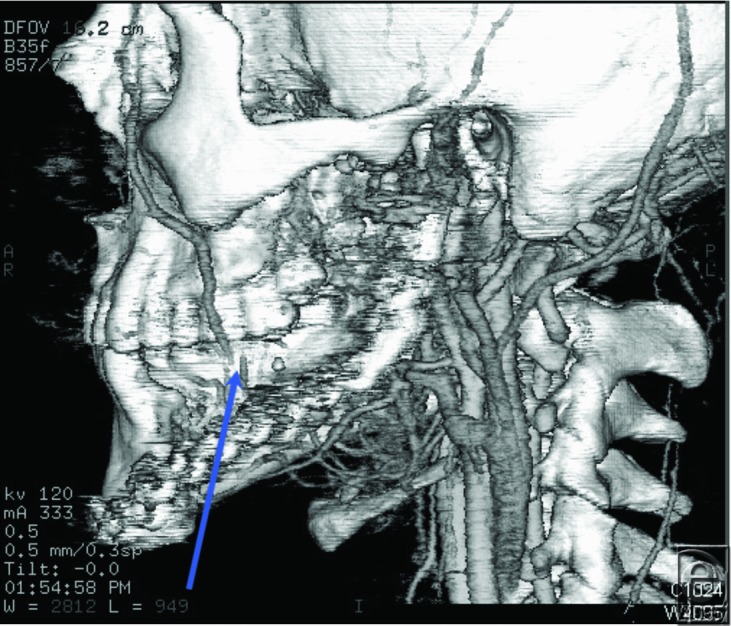
CTA of recipient site for head and neck reconstruction. CTA of head and neck vessels in a patient with a fractured mandibular reconstruction plate. There is total occlusion of the facial artery and significant soft tissue damage near the external carotid artery.

A 22-year-old man with a history of left mandibular ameloblastoma was considered for a free fibula osteocutaneous flap reconstruction after fracturing his primary mandibular reconstruction plate. A CTA of his left head and neck vessels revealed total occlusion of the facial artery and significant soft tissue damage near the external carotid artery, likely due to his previous surgery and radiation treatment (Fig 4). The patient did not desire access into his contralateral neck for microvascular reconstruction and received a replacement mandibular reconstruction plate instead.

### Case 5: CTA of donor site in head and neck reconstruction

**Figure 5:**
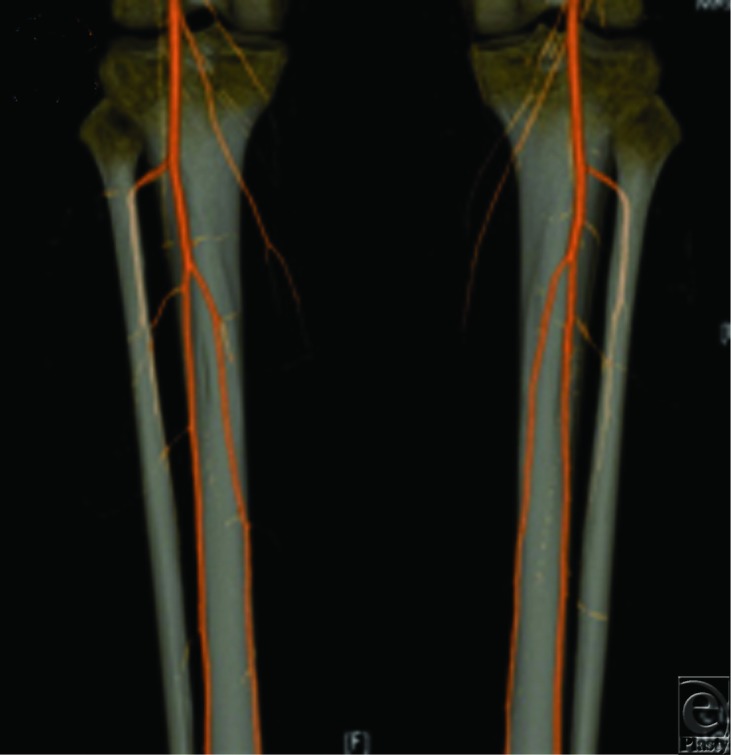
CTA of donor site for head and neck reconstruction. CTA of bilateral lower extremities in a patient with ameloblastoma of the mandible demonstrating bilateral peroneus magnus.

A 41-year-old woman with ameloblastoma of the mandible presented for free fibula flap reconstruction. On examination, dorsalis pedis and posterior tibial pulses were weakly palpable. A preoperative CTA of the lower extremities demonstrated bilateral peroneus magnus (Fig 5). Therefore, the patient successfully underwent a free iliac crest flap instead.

## DISCUSSION

Computed tomographic angiography has been lauded as an excellent alternative to traditional angiography because it is less invasive, involves less radiation exposure, and is considerably more cost-effective.^11^ One of the main arguments against routine arteriography is the considerable rate of major complications, which has been reported to be as high as 7%.^17^ Numerous studies have demonstrated the safety of CTA, and our results further confirm this conclusion. We observed no CTA-related complications in our patient population.

The use of CTA has been documented in the vascular medicine literature, and a number of studies have highlighted its potential use in plastic surgery. Computed tomographic angiography has been used to identify zones of perfusion^18^^,^^19^ and to aid in the intraoperative dissection of free abdominal flaps in breast reconstruction.^20^ Preoperative CTA was found to be highly accurate in mapping the perforators of the deep inferior epigastric artery^21^^-^23 and was associated with decreased operative times, flap complications, and donor site morbidity.^24^^,^^25^

Our study shows that CTA is reliable in its ability to detect vascular anomalies that influence the planning of various microsurgical reconstructive procedures (Table 3). These results are similar to other studies in which CTA was performed for mapping of atherosclerotic lower limb vasculature.^26^^,^^27^ Recent studies corroborate these findings for CTA used in plastic surgery applications for the anterolateral thigh flap harvest^14^^,^^28^ and abdominal-based free flaps used for breast reconstruction.^15^

Preoperative vascular imaging at the free flap recipient site is often obtained when there has been significant trauma or prior extensive surgery. Routine imaging of the donor site is somewhat controversial. There is evidence that clinical examination of distal pulses in fibula flap evaluation is as accurate as traditional angiography while safer and more cost-effective.^29^^-^31 Computed tomographic angiography, however, not only offers a volumetric display of the vasculature but also allows the surgeon to see the desired vascular anatomy in relationship to the surrounding soft tissues. This study is the first of sufficient size to document vascular abnormality rates detected by CTA in patients undergoing reconstructive surgery of all types.

We report a total abnormality rate of 51%. This number at first blush may seem high, but one must consider our particular patient population. It is important to note that the vast majority of patients receiving free flaps who also received a CTA were considered a high-risk group with complex reconstructive issues, comorbidities, and concerns for possible abnormal vascular anatomy. Hence, our study includes a selected population, which, in part, explains the high incidence of vascular anomaly findings on CTA. Nonetheless, an abnormality rate of 51% during preoperative CTA is large enough to warrant attention. Most abnormality rates reported in the plastic surgery literature using traditional angiography or clinical pulse examination are considerably lower. These differences may be attributed to the better spatial resolution of CTA and particularly to its ability to delineate soft tissue injuries that cannot be detected with traditional angiography.

Vascular abnormalities detected as a result of preoperative CTA caused changes in operative plans in 45% of patients (Table 2). Changes included use of alternate recipient vessels, use of pedicled flaps instead of free flaps, or harvest of free flaps from the contralateral side. Furthermore, surgeons were able to work around known soft tissue injuries and decide on the location of the anastomosis using patency data from CTA as well as direct vessel visualization. Of note, obtaining CTA was considerably less expensive than traditional angiography with an average cost at our institution of $1140 as compared with $3900 for traditional angiography.^11^

Our study identified some limitations of CTA, especially in the presence of external metal fixators (Table 3). Although CTA provides the ability to rotate images 3-dimensionally, artifact created by metallic hardware can make it difficult to reliably delineate the vascular anatomy. However, current improvements in CTA technology are underway to suppress the metal-induced aberrations. Another drawback of CTA is that it requires at least as much contrast load as traditional angiography, which limits its use in patients with impaired renal function. Radiation is also a concern with any additional CT scan.^32^ Currently, MRA is under investigation to provide imaging without radiation, but studies are small and have mixed results for plastic surgery applications.^33^^,^^34^ In addition, any patient with a pacemaker or noncompatible metal implant would be eliminated from MRA evaluation.

Our study shows that CTA is a safe and reliable tool for delineating the vascular anatomy prior to microsurgical reconstruction (Table 3). Preoperative CTA may find its greatest utility in situations where there is concern or ambiguity about the vascular anatomy. In our series, 51% of the scans revealed some vascular abnormality. The operative plan was modified on the basis of CTA results in 45% of all patients (88% of patients with abnormal CTA findings). However, not all anatomic sites had the same rate of CTA abnormalities. Abnormality rates in the breast and chest wall were less than 33%, in the lower extremity between 33% and 66%, and in the upper extremity greater than 66% (Table 2). These differences can be explained in part by the etiology of the disease process. For example, CTA of the upper extremity was mainly performed in trauma cases in which there was a 55% incidence of abnormal findings as compared with infection (45%) or cancer (43%).

The degree of CTA influence on operative planning varied considerably with the type of surgery performed. Reconstructions were more likely to be influenced by CTA after trauma or cancer than after infection (Table 2).

Our results serve as a basis upon which recommendations for preoperative CTA can be made. The use of CTA should be considered for cases of microsurgical reconstruction where the vascular anatomy may be in question. This was most often the case for traumatic or infectious injuries of the upper extremity, for fibula flap donor sites, and injuries of the lower extremities. The plastic surgeon is advised to have a low threshold for obtaining a CTA in those cases. However, for cases where the vascular anatomy has shown to be fairly consistent or where there is no trauma and no potential cause for vascular distortion, the utility of CTA may decrease. This was the case for breast or chest reconstructions, for which preoperative CTA is not recommended unless extensive surgery, scarring, or prior radiation is present. Further studies are warranted to evaluate the cost-effectiveness of routine CTA for all cases of reconstruction.

## CONCLUSIONS

Computed tomographic angiography is a safe and reliable method of preoperatively assessing vessels used in microsurgical reconstruction. When an abnormality in vascular anatomy is detected by CTA, the surgeon is advised to consider altering the operative plan accordingly. Choosing alternate vessels to use as recipients or changing the method of reconstruction may be necessary to avoid vascular complications. Computed tomographic angiography may not be able to fully delineate the vascular anatomy when there is significant metallic artifact present such as an external fixation device, but technological advancements are improving CTA resolution in these circumstances.

## Figures and Tables

**Table 1:** Patient demographics*

Patients, n		94
Age, mean ± SD, y		39 ± 19
Gender		
	Male	55 (59%)
	Female	39 (41%)
Planned free flap		
	Anterolateral thigh	2 (2%)
	Fibula	18 (19%)
	Gracilis	8 (9%)
	Great Toe	1 (1%)
	Lateral arm	2 (2%)
	Latissimus	15 (16%)
	Rectus	17 (18%)
	Radial Forearm	13 (14%)
	TRAM	14 (15%)
Indication for CTA		
	Upper extremity trauma	20 (21%)
	Lower extremity trauma	34 (36%)
	Free fibula donor site	18 (19%)
	Head and neck recipient site	6 (7%)
	Breast recipient site	13 (14%)

CTA, computed tomographic angiography; SD, standard deviation; TRAM, transverse rectus abdominus myocutaneous.

*Values are expressed as n (%) unless otherwise indicated.

**Table 2:** CTA influences planning of microsurgical reconstruction

CTA site	No. of cases	Abn CTA	Plan changed	Changed/Abn
UE	21	86%	86%	100%
LE	34	41%	29%	71%
Free fibula	17	41%	41%	100%
Head & Neck	8	63%	38%	60%
Breast/Chest wall	13	23%	23%	100%
Abdomen	1	100%	100%	100%
Donor	17	41%	41%	100%
Recipient	77	53%	45%	85%
Totals	94	51%	45%	88%
Trauma	44	55%	50%	92%
Cancer	35	43%	43%	100%
Infection	11	45%	27%	60%
Other	4	100%	100%	50%

Abn indicates abnormal; CTA, computed tomographic angiography; LE, lower extremity; UE, upper extremity.

**Table 3:** CTA properties and complications

Statistical properties of CTA		
	Sensitivity	94%
	Specificity	97%
	Positive predictive value	97%
	Negative predictive value	95%
Quality of images		
	Clear	95%
	Vessel obstruction by metal fixator	3%
	Excessive venous flow	1%
	Extensive soft tissue distortion	1%
Complications		
	CTA complications	0%
	Flap failure rate	1%

CTA indicates computed tomographic angiography.
